# “Down the Rabbit Hole” of Vaccine Misinformation on YouTube: Network Exposure Study

**DOI:** 10.2196/23262

**Published:** 2021-01-05

**Authors:** Lu Tang, Kayo Fujimoto, Muhammad (Tuan) Amith, Rachel Cunningham, Rebecca A Costantini, Felicia York, Grace Xiong, Julie A Boom, Cui Tao

**Affiliations:** 1 Department of Communication Texas A&M University College Station, TX United States; 2 School of Public Health The University of Texas Health Science Center at Houston Houston, TX United States; 3 School of Biomedical Informatics The University of Texas Health Science Center at Houston Houston, TX United States; 4 Immunization Project Texas Children’s Hospital Houston, TX United States; 5 Department of Neuroscience University of Texas Austin, TX United States

**Keywords:** vaccine, misinformation, infodemiology, infodemic, YouTube, network analysis

## Abstract

**Background:**

Social media platforms such as YouTube are hotbeds for the spread of misinformation about vaccines.

**Objective:**

The aim of this study was to explore how individuals are exposed to antivaccine misinformation on YouTube based on whether they start their viewing from a keyword-based search or from antivaccine seed videos.

**Methods:**

Four networks of videos based on YouTube recommendations were collected in November 2019. Two search networks were created from provaccine and antivaccine keywords to resemble *goal-oriented browsing*. Two seed networks were constructed from conspiracy and antivaccine expert seed videos to resemble *direct navigation*. Video contents and network structures were analyzed using the network exposure model.

**Results:**

Viewers are more likely to encounter antivaccine videos through direct navigation starting from an antivaccine video than through goal-oriented browsing. In the two seed networks, provaccine videos, antivaccine videos, and videos containing health misinformation were all found to be more likely to lead to more antivaccine videos.

**Conclusions:**

YouTube has boosted the search rankings of provaccine videos to combat the influence of antivaccine information. However, when viewers are directed to antivaccine videos on YouTube from another site, the recommendation algorithm is still likely to expose them to additional antivaccine information.

## Introduction

### Background

The proliferation of social media has allowed the antivaccine movement to become more influential than at any point in history [1]. Earlier studies have demonstrated that social media platforms such as Pinterest and Twitter are filled with antivaccine information [2,3]. The consumption of antivaccine social media content could negatively impact vaccine attitudes and consequently vaccine uptake [4]. Furthermore, social media can indirectly influence the public by setting the agenda of traditional mass media in vaccine-related controversies [5]. In some cases, social media platforms such as Twitter have even been weaponized to promote antivaccine messages through the use of bots and trolls [6].

YouTube is the largest video-sharing platform in the world with more than 1 billion users. However, it is a hotbed for antivaccine information. Researchers examined 172 YouTube videos related to the human papillomavirus vaccine and concluded that only slightly over 30% of the videos were provaccine [7]. A more recent study of influenza and measles-mumps-rubella vaccine videos on YouTube showed that around 20% and 30% of the videos were antivaccine in nature, respectively [8]. In both studies, antivaccine videos received more views and likes than provaccine videos [7,8].

YouTube has come under criticism because its recommendation algorithm keeps viewers watching videos by suggesting similar videos based on their viewing histories. In other words, YouTube creates filter bubbles where viewers are exposed to repetitive, homogenous, and often biased content, which further reinforces biases and misconceptions. Scholarly attention has been paid to YouTube content promulgating politically extreme ideologies [9], but little is known about the spread of harmful health content such as misinformation about vaccines on this platform. In this study, we jump into the “rabbit hole” driven by YouTube’s recommendation algorithm to explore viewers’ exposure to vaccine-related information and misinformation to determine to what extent, if any, YouTube’s search and recommendation algorithms impact the information to which audiences are exposed.

### Diffusion of Information on YouTube

Traditionally, scholars have explained how individuals consume media information using the selective exposure paradigm, which predicts that individuals tend to select media content consistent with their existing beliefs and attitudes [10]. In the era of social media, individuals’ exposure to social media content is largely influenced by the recommendations of their friends or the celebrities they follow or subscribe to [11]. However, one’s friends and associates on social media tend to share similar interests and opinions; consequently, relying on the recommendation of one’s contacts is likely to create echo chambers, where one is exposed to conforming opinions repeatedly [11]. In addition, individuals’ consumption of social media content is influenced by the machine learning–based recommendation algorithms of the platform. Such personalization of content creates filter bubbles, in which algorithms recommend information that users have previously been exposed to and with which they agree [11]. Empirical studies have shown that both echo chambers and filter bubbles deepen the ideological divide among the public [11].

### Information Exposure on YouTube

Users interact with the YouTube platform in two ways. First, *direct navigation* occurs when users are directed to watch a YouTube video from another website or social media platform. Alternatively, users could search for videos based on keywords (*goal-oriented browsing*) [12]. In both cases, YouTube will present users with a set of recommended videos based on the user’s prior viewing behaviors as well as covisitation counts [13]. To understand the type of vaccine information YouTube users are exposed to when they use direct navigation or goal-oriented browsing, we propose the first research question (RQ1): When YouTube users start their viewing with provaccine or antivaccine keywords, or an antivaccine seed video, to what extent will they will be exposed to pro- and antivaccine content?

**Figure 1:**
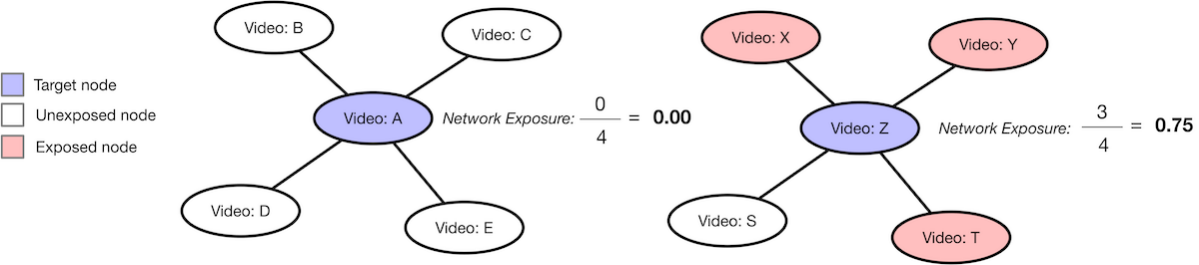
Network exposure model example showing two nodes (A and Z) exposed to attributes based on their ties. The example was adapted from Valente [15].

Thus, RQ1 explores YouTube users’ exposure to pro- and antivaccine videos on a macro level when they use YouTube in different ways; however, it is also important to examine the connections among different types of videos in recommendation networks on a micro level. Based on the diffusion of innovation theory [14], the network exposure model (NEM) measures the degree to which a node in the network is exposed to other nodes with a certain attribute. A node’s exposure to an attribute is computed from the average edge from a node to other nodes that exhibit the attribute. In Figure 1 (adapted from Valente [15]), we present two examples with nodes that have connections to other nodes. Some of the nodes (representing any type of entity such as a YouTube video) in Figure 1 are color-coded in pink to indicate that it has an attribute (such as containing misinformation). In Figure 1, Video A has an exposure value of 0 because it has no connections to nodes containing misinformation (ie, 0/4=0.00). In comparison, Video Z has a network exposure value of 0.75, because three out of four edges connect it to nodes (pink nodes) containing misinformation (ie, 3/4=0.75). A more detailed description of the metric can be found in the related literature [15-18].

Overall, the NEM measures the extent to which one node in the network is exposed to a certain type of node. To understand how likely provaccine and antivaccine videos, as well as other types of videos unrelated to vaccines, are to lead to antivaccine information, we addressed RQ2: What is the degree of exposure of pro- and antivaccine videos as well as other videos unrelated to vaccines to additional antivaccine videos?

## Methods

### Data Collection and Network Generation

To collect YouTube videos based on a keyword search (*goal-oriented browsing*), we used four provaccine key phrases (“why I vaccinate,” “vaccinate with me,” “vaccine saves lives,” and “vaccine works”), and four antivaccine key phrases (“vaccine causes autism,” “vaccine kills,” “vaccine takes life,” and “vaccine harm”). These key phrases were based on the most popular positive and negative hashtags about vaccines on Twitter in October 2019. We utilized CAS^2^T [19], an open-sourced tool that leverages the YouTube application programming interface (API) [20] to create networks of related YouTube videos and retrieve each video’s recommended videos. The API does not factor in the viewing histories of individual users and only retrieves videos based on YouTube recommendation algorithms. This tool also stores the collections of videos and their recommended videos in a relational database (SQLite), along with their metadata information (ie, views, likes). For the two networks based on provaccine and antivaccine key phrases (“search networks”), we collected the first 6 videos’ URLs and fetched three depth levels of related videos. Through this procedure, we gathered 6 related videos for each of the videos, and then the related videos’ related videos, and so on. We presumed that users might not view more than 6 recommended videos due to the screen size of typical computers and the number of recommended videos shown on one screen. We performed the same procedure with the videos of referral sharing (“seed network”) for vaccine-based conspiracy videos (ie, “big pharma money-making scheme,” “government covering up side-effects,” “distrust of doctors”) and antivaccine experts claiming authority on the topic of vaccines. The *conspiracy seed network* was seeded with 16 videos and the *antivaccine expert seed network* was seeded with 8 videos. Seed videos were sourced from two playlists on their respective topics. Thus, this provided a good start to further assess the misinformation rabbit hole through referral sharing. Data collection was conducted in November 2019. After data collection, we aggregated the databases from CAS^2^T into one central database repository using PostgreSQL v12, and assigned a unique identification number to each video.

### Annotation

To annotate the two search networks, we started with 815 videos that were initially downloaded. After deleting replicated videos, a total of 538 videos were annotated. We annotated these videos in terms of (1) whether the video was related to vaccines and (2) if the video was related to vaccines, whether it was provaccine or antivaccine. For videos that were unrelated to vaccines, we annotated (1) whether it was about autism and (2) whether it contained other health information and misinformation. Three of the authors (LT, RAC, FY) annotated 54 videos (10%) selected through random systematic sampling and achieved excellent intercoder reliability according to Krippendorff α values (related to vaccine, α=.949; pro- or antivaccine, α=.90; containing autism-related information, α=.96; and containing other health misinformation, α=.949). The three authors then split all of the videos and annotated them independently. Videos in a language other than English were identified as such and excluded from data analysis. We annotated the two seed networks (conspiracy and antivaccine expert networks) using the guidelines described at the beginning of this subsection. Two of the authors (TA, GX) annotated the 1034 seed-based videos, demonstrating excellent reliability (vaccine-related, α=.899; health-based, α=.901; autism-related, α=.96; and misinformation, α=.806). Videos related to vaccines and that contained misinformation were then recoded as “antivaccine videos” and other vaccine-related videos were recoded as “provaccine videos” to create consistency between the coding results of the two groups.

### Data Analysis

We analyzed the four networks using various network metrics to understand the relationships among different types of videos within their respective networks. CAS^2^T conveniently generates tables for node metadata, nodes, and edges, which, for the nodes and edges, can be seamlessly imported into Gephi to construct undirected networks to perform network analysis. Gephi [21] was used to compute the network statistics and generate the visualizations of the networks.

For each of the four networks, we examined how likely nonvaccine videos, vaccine-related videos (provaccine and other antivaccine videos), autism videos, and health-related videos (unrelated to vaccines) are likely to be exposed to antivaccine videos. We used NET-EXPO [22], a Gephi plugin, to compute network exposure. STATA v15 and SPSS v26 were used for basic statistical calculations and frequency computations. We calculated the case-control odds ratio to measure how likely different types of videos (provaccine videos, antivaccine videos, autism videos unrelated to vaccines, health videos unrelated to vaccines, and health misinformation unrelated to vaccines) were exposed to antivaccine videos.

## Results

Four network datasets were generated based on provaccine and antivaccine search key phrases (“search network”) and on conspiracy and antivaccine expert seed videos (“seed network”). Each node in these networks represents a video and each edge represents a recommendation relationship (see Table 1 for the descriptive statistics of the four networks). The node size ranged from 283 to 551, and the number of edges ranged from 342 to 671. The average degree for all of the networks was approximately 2.4, except for the conspiracy network, which was 2.3. The average clustering coefficient, which measures the clustering of the nodes in a network, was 0.415 and 0.411 for provaccine and antivaccine search networks respectively, and was 0.06 and 0.1 for the conspiracy seed network and antivaccine expert seed network, respectively.

**Table 1:** Global statistics for the four collected networks.

Characteristic		Search networks		Seed networks	
		Provaccine search network	Antivaccine search network	Conspiracy seed network	Antivaccine expert seed network
**Network characteristics**					
	Nodes, n	283	354	483	551
	Edges, n	342	417	551	671
	Average degree	2.4	2.4	2.3	2.4
	Network diameter	8	8	14	12
	Average clustering coefficient	0.415	0.411	0.06	0.1
**Video type, n (%)**					
	Nonvaccine-related	242 (86)	315 (89)	413 (86)	511 (93)
	Vaccine-related	41 (14)	40 (11)	70 (14)	40 (7)
	Provaccine^a^	38 (93)	35 (87.5)	34 (49)	15 (38)
	Antivaccine^a^	3 (7)	5 (12.5)	36 (51)	25 (63)
**Source of videos, n (%)^a^**					
	Government agencies	23 (56)	14 (35)	0 (0)	0 (0)
	Academic institutions and hospitals	6 (15)	13 (33)	9 (13)	1 (3)
	Pharmaceutical companies and for-profit organizations	1 (2)	0 (0)	1 (1)	0 (0)
	Consumer-generated	3 (7)	5 (13)	33 (47)	26 (65)
	News media	8 (20)	9 (23)	27 (39)	13 (33)
	Professional associations	0 (0)	2 (5)	0 (0)	0 (0)
	Other	0 (0)	2 (5)	0 (0)	0 (0)
Autism-related video, n (%)		6 (2)	21 (6)	13 (3)	22 (4)
Health-related video, n (%)		100 (35)	142 (40)	267 (55)	316 (57)
Accurate health information, n (%)^b^		99 (99)	139 (98)	157 (59)	172 (54)
Health misinformation, n (%)^b^		1 (1)	3 (2)	110 (41)	144 (46)

^a^Percentages are based on the number of vaccine-related videos in a given network.

^b^Percentages are based on the number of health-related videos in a given network.

RQ1 asked whether starting with provaccine and antivaccine keywords, and starting from antivaccine seed videos will lead viewers to pro- or antivaccine information. In the two search networks generated from provaccine and antivaccine keywords, an overwhelming majority of the vaccine-related videos were provaccine (Table 1). Most of these vaccine-related videos were created and uploaded by credible sources such as governmental agencies, hospitals, and academic institutions. In contrast, viewers were much more likely to be exposed to antivaccine information in the conspiracy seed network and in the antivaccine expert seed network. Approximately half of the vaccine-related videos in the two seed networks were consumer-generated (Table 1).

**Figure 2:**
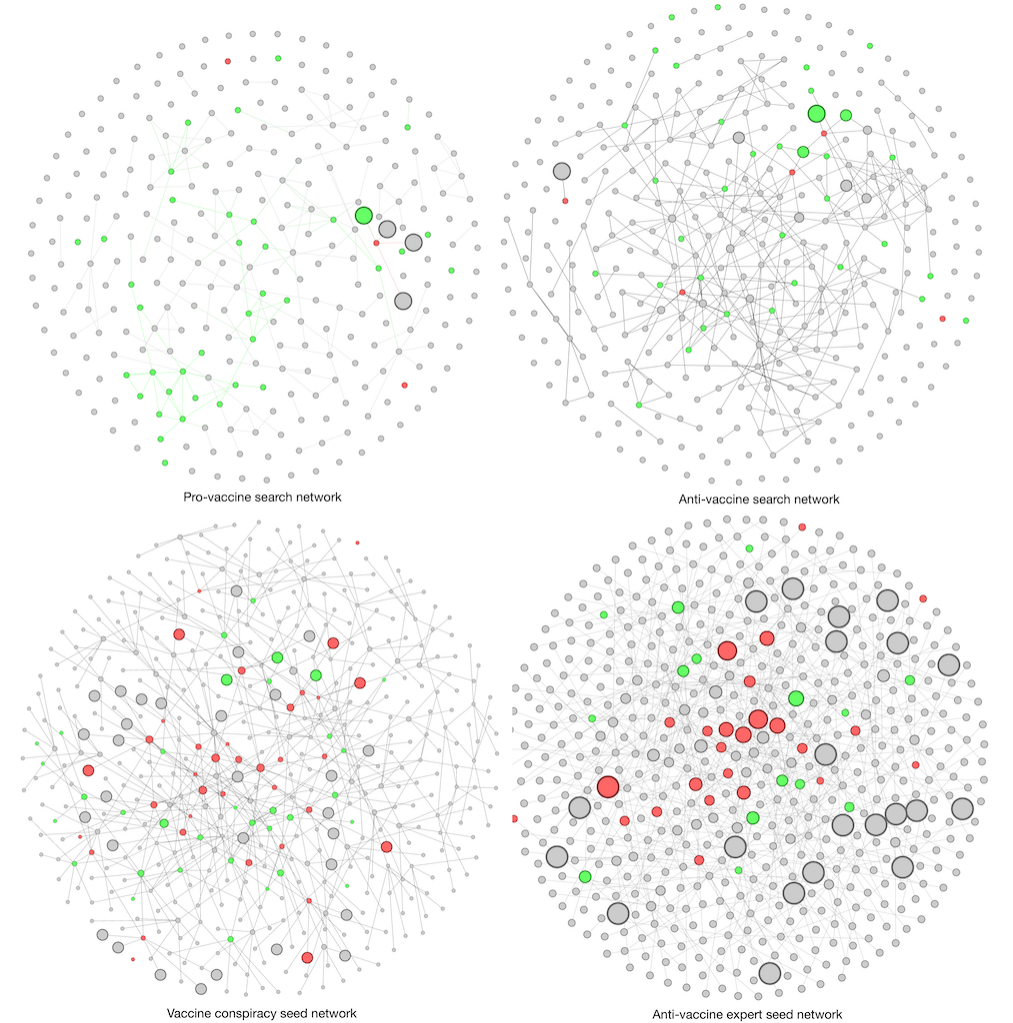
Visualization of the four YouTube video networks, where each node represents a video and an edge represents a related link. Green represents a provaccine video and red represents an antivaccine video. Gray nodes are nonvaccine-related videos. The size of the nodes depicts the exposure value to antivaccination videos. Visualization was generated with Gephi.

RQ2 examined the degree of exposure of different types of videos to additional antivaccine information. Table 2 presents the odds ratios for exposure to antivaccine misinformation and Figure 2 provides visualizations of the four networks.

In the search networks, nonvaccine videos had a low likelihood of being exposed to antivaccine videos within their respective networks (provaccine and antivaccine search networks). However, vaccine-related videos had a higher chance of being exposed to antivaccine videos. Within the vaccine-related videos of the search networks, provaccine video nodes were more likely to be exposed to antivaccine videos in both networks. However, none of these odds ratios was statistically significant. The only significant results in these two networks were found for the antivaccine search network, autism videos, and health-related videos (whether contentious or noncontentious), which had no chance of being exposed to antivaccine videos. However, the picture was very different in the two seed networks.

In seed networks, compared to vaccine-related videos, videos unrelated to vaccines had a significantly lower chance of being exposed to antivaccine videos (for both the conspiracy seed network and antivaccine expert seed networks). Antivaccine videos had tremendous exposure to other antivaccine videos (in both the conspiracy seed network and antivaccine expert seed network). In comparison, provaccine videos were also vulnerable to antivaccine video recommendations, but to a lesser extent. Furthermore, videos containing nonvaccine health misinformation had greater odds of being exposed to antivaccine videos in both the conspiracy seed network and the antivaccine expert seed network. In comparison, videos containing accurate health information did not exhibit this pattern.

**Table 2:** Descriptive statistics of the exposure to antivaccine video nodes.

Statistic		Search networks		Seed networks	
		Provaccine search network	Antivaccine search network	Conspiracy seed network	Antivaccine expert seed network
Mean (SD)		0.01 (0.12)	0.02 (0.10)	0.12 (0.28)	0.07 (0.21)
Range		1-1	0.11-1	0.13-1	0.13-1
Nodes exposed, n (%)		4 (1.4)	15 (4.2)	119 (24.7)	86 (15.6)
Nodes unexposed, n (%)		279 (98.6)	339 (95.8)	364 (75.3)	465 (84.4)
**Odds ratio (95% CI)**					
	Nonvaccine video	0.50 (0.04-27.0)	0.48 (0.12-2.8)	0.07 (0.04-0.14)	0.04 (0.02-0.09)
	Vaccine video	1.99 (0.04-25.0)	2.1 (0.36-8.3)	13.6 (7.3-25.9)	24.4 (10.8-58.4)
	Provaccine video	2.18 (0.04-27.9)	2.4 (0.41-9.5)	8.94 (3.9-21.6)	12.1 (3.6-46.1)
	Antivaccine video	0.00 (0-108.7^a^)	0.00 (0.0-18.1^a^)	11.6 (5.0-28.8)	27.9 (9.6-97.3)
	Autism video	0.00 (0-50.3^a^)	0.00 (0.0-4.0^a^)	0.92 (0.16-3.6)	2.1 (0.65-5.9)
	Health video	5.62 (0.44-297)	0.00 (0.0-0.36^a^)	1.52 (0.98-2.4)	2.0 (1.2-3.5)
	Accurate health information	5.71 (0.45-301.8)	0.00 (0.0-0.37^a^)	0.97 (0.60-1.5)	1.22 (0.72-2.0)
	Health misinformation	0.00 (0.00-0.00)	0.00 (0.0-30.6^a^)	1.80 (1.1-2.9)	1.76 (1.0-2.9)

^a^Cornfield exact CI.

## Discussion

### Principal Findings

This study tested the characteristics of YouTube’s search and recommendation algorithms by exploring the information users are likely to be exposed to when they begin with keyword-based searches and when they start from an antivaccine seed video. We utilized network exposure models, along with other statistical methods, to determine how vulnerable the videos (and by proxy, the users) are to antivaccine content. Four networks—provaccine search network, antivaccine search network, conspiracy seed network, and antivaccine expert seed network—were examined in terms of both video content and network structures.

First, when users start with a keyword-based search on YouTube, they are likely to reach provaccine videos posted by credible sources such as government agencies and hospitals, regardless of whether they used provaccine or antivaccine keywords. This encouraging finding suggests that YouTube has taken some measures to promote provaccine videos from credible sources in their search function. This contradicts prior research that reported that antivaccine videos appear in the top search hits [23]. Antivaccine users such as Alex Jones, InfoWars, and the like have been banned from YouTube, taking with them their controversial and extreme content [24]. Although certain major players of the antivaccine movement have been removed, there were still several questionable “experts” that emerged in our network data. These antivaccine experts use misleading rhetoric about vaccines that may sound plausible to naïve viewers. Even when such antivaccine experts’ videos might not be easily found through keyword searches, they can be found and shared with a network of friends through email or social media, directing the viewers to the proverbial “rabbit hole” of misinformation.

Second, even if users are to watch a provaccine video, they have a relatively high chance of being recommended an antivaccine video. A viewer could have a roughly 2 to 12 times chance of being recommended an antivaccine video depending on the networks than any other video. Antivaccine videos are much more likely to lead to more antivaccine videos. It should be noted that such patterns were statistically significant in the two seed networks but not in the two search networks, which is likely to be caused by the extremely low percentages of antivaccine videos in the two search networks. Furthermore, compared with vaccine-related videos, nonvaccine videos were more isolated from antivaccine content in all four networks. It is assumed that YouTube uses a machine-learning algorithm that looks at titles, descriptions, and other metadata, and matches them with similar data in other videos to denote recommended videos. Therefore, it may not be surprising that nonvaccine videos are somewhat isolated from vaccine videos and that antivaccine videos beget more antivaccine videos.

Third, we found that 2%-6% of the videos in these networks were about autism but did not contain any vaccine-related information. This means that when users watch autism-related videos, they might be directed to vaccine-related videos or even to antivaccine videos. This could potentially contribute to the spread of misinformation about the vaccine-autism link. Furthermore, health videos that discuss nonvaccine health topics (eg, diet, holistic medicine, cancer), especially those that contain misinformation, have some vulnerability to antivaccine videos through recommendations. In the conspiracy and antivaccine expert seed networks, a viewer may come across an antivaccine video while watching a health-related video containing other types of misinformation.

### Limitations and Future Directions

One limitation of the current study is the use of the YouTube API. Due to its proprietary nature, it is difficult to ascertain how and why YouTube recommends videos. It is assumed that such recommendations are based on a combination of machine-learning approaches that factor in video metadata, the similarity of words in titles and descriptions with other videos, user preferences, and viewing history. Our networks were based on YouTube recommendations without personal viewing history. In reality, recommended videos may vary with viewers’ history and preferences that are included in the calculation. At best, the current study provides a baseline that does not factor in users’ viewing history or other preferences. In addition, we came across a considerable number of videos with health information unrelated to vaccines in the networks. It appears that misinformation will lead to additional misinformation. Thus, future research could investigate the content that propagates fad diets, questionable food supplements, and other unsubstantiated health information on YouTube.

### Conclusions

This study explored the information YouTube users are exposed to when they start with search keywords or start with seed videos through social network analysis. Utilizing the NEM, we examined the odds of vaccine misinformation propagated to users through individual nodes. Our results showed that although vaccine misinformation is sandboxed within a network of vaccine videos, some provaccine videos are susceptible to antivaccine videos through YouTube recommendations. There is also evidence that health-related videos, especially those containing health misinformation, are vulnerable to vaccine misinformation. Overall, individuals watching YouTube videos through a goal-oriented search have a lower chance of encountering vaccine misinformation due to efforts from YouTube; however, antivaccine misinformation still exists and users have a chance to encounter these videos or other misinformation content through direct navigation.

## Abbreviations

API: application programming interface
NEM: network exposure model
